# Right heart bypass without an oxygenator as an alternative technique for surgical management of right-sided heart problems: a case report

**DOI:** 10.1097/MS9.0000000000003219

**Published:** 2025-04-10

**Authors:** Mehrzad Rahmanian, Maede Mohammadian, Reza Mollazadeh, Mani Moayerifar, Maziar Moayerifar, Mahboobeh Gholipour, Zahra Ahmadi Hasanabad

**Affiliations:** a Department of Cardiovascular Surgery, Imam Khomeini Hospital Complex, Tehran University of Medical Sciences, Tehran, Iran; b Department of Radiology, Shohada-e-Tajrish Hospital, Shahid Beheshti University of Medical Sciences, Tehran, Iran; c Department of Cardiology, Imam Khomeini Hospital Complex, Tehran University of Medical Sciences, Tehran, Iran; d Department of Cardiology, Healthy Heart Research Center, Heshmat Hospital, School of Medicine, Guilan University of Medical Sciences, Rasht, Iran; e Department of Vascular Surgery, Razi Hospital, Guilan University of Medical Sciences, Rasht, Iran

**Keywords:** case report, implantable cardioverter defibrillator, infective endocarditis, right heart bypass

## Abstract

**Introduction:**

Lead-related infective endocarditis is a rare complication with a high mortality rate that needs to be addressed immediately. In most cases, surgery is mandatory in addition to medical treatment. Alternative techniques to conventional cardiopulmonary bypass have been developed to reduce the inflammatory response and post-operative complications.

**Case presentation:**

A 29-year-old male patient with a dual-chamber implantable cardioverter defibrillator presented to our hospital with infective endocarditis. Early evaluation of the patient revealed a large mobile 6*2 cm vegetation attached to the leads and tricuspid valve and the right atrium. Due to large size of the vegetation, the patient got prepared for surgical removal of the vegetation. The patient underwent right heart bypass with cannulation of the inferior and superior vena cava and the pulmonary artery.

**Discussion:**

The inflammatory response subsequent to cardiopulmonary bypass plays an important role in postoperative complications such as pulmonary complication including transient hypoxemia or even more severe complication like acute respiratory distress syndrome. Additionally, the ascending aorta manipulation may result in postoperative cerebral atheroembolism and stroke. In selected patients, right heart bypass is a better option, as cardioplegic arrest is not needed in this technique, and a less post-operative inflammatory response is expected due to the absence of long tubes, an oxygenator, and aortic clamping.

**Conclusion:**

Right heart bypass could be a less complicated, safer, and cost-benefit alternative to conventional cardiopulmonary bypass in the case of right-sided heart problems. This technique allows effective decompression of the right heart and adequate oxygenation without postoperative complications, usually seen in routine cardiopulmonary bypass.

## Introduction

Lead-related endocarditis is a rare complication with a high mortality rate that needs to be addressed immediately^[^1^]^. In most cases, surgery is mandatory in addition to medical treatment. Although cardiopulmonary bypass is a revolution in cardiac surgery, alternative techniques have been developed to reduce the inflammatory response and post-operative complications^[^2^]^. In this article, we present a patient with implantable cardioverter defibrillator (IDC)-related infective endocarditis who underwent right heart bypass for removal of the vegetation. The work has been reported in line with the Surgical CAse REport (SCARE) criteria^[^3^]^.

**HIGHLIGHTS**

- Lead-related endocarditis is a rare complication with a high mortality rate that needs to be addressed immediately.
- In most cases, surgery is mandatory in addition to medical treatment.
- Although cardiopulmonary bypass is a revolution in cardiac surgery, alternative techniques have been developed to reduce the inflammatory response and post-operative complications.
- Right heart bypass could be a less complicated, safer, and cost-benefit alternative to conventional cardiopulmonary bypass in the case of right-sided heart problems.

## Case presentation

**Figure 1.**
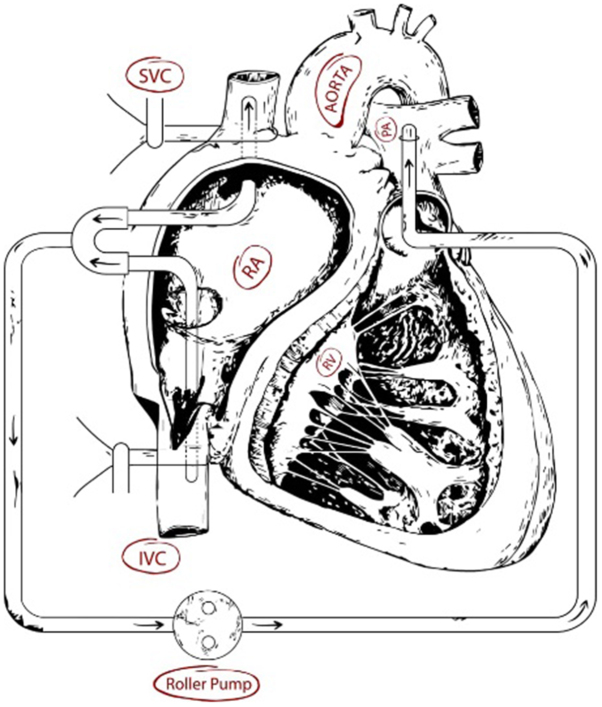
The right heart bypass established between a 21 F aortic cannula inserted into the main pulmonary artery and two 28 F cannulas inserted into the SVC and IVC. Because of the high pulmonary arterial pressure, a roller pump placed between the main pulmonary artery and the SVC and the IVC cannulas to maintain the sufficient blood flow to the pulmonary artery.

**Figure 2.**
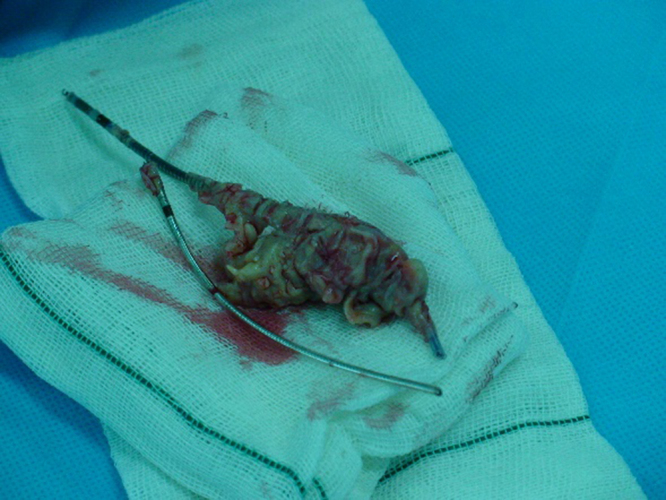
The post-operative photograph of 5.6*2.1 cm vegetation attached to ICD leads.

A 29-year-old male patient with ICD presented to our hospital emergency department (ED) with fever, chills, and shortness of breath. The patient had a history of emergent thoracotomy and laparotomy due to multiple trauma after a car accident a year ago. After the thoracotomy, the patient was diagnosed with a low heart ejection fraction; therefore, a dual-chamber ICD was implanted for him. At ED, the vital signs included a blood pressure of 100/60 mmHg, a pulse rate of 96 beats/min, a respiratory rate of 24 breaths/min, and an axillary temperature of 38.8°C, and his pulse oximetry oxygen saturation was 97%. The blood tests revealed leukocytosis and an elevated level of erythrocyte sedimentation rate and C-reactive protein. The early evaluation of the patient with transesophageal echocardiography revealed a large mobile 6*2 cm vegetation attached to the ICD leads and tricuspid valve and the right atrium. Since the medical management failed and due to a large size of the vegetation, the patient got prepared for urgent operation. Preoperative transthoracic echocardiography showed mild-to-moderate tricuspid regurgitation, and the pulmonary arterial pressure (PAP) was estimated to be 35 mmHg. In the operating room, a median sternotomy was performed, and heparin (3 mg/kg) was administered 3 minutes before the initiation of right heart bypass (RHB). During the operation, the tidal volume was decreased to facilitate the surgeon’s work and the rate was increased to maintain the proper amount of arterial blood gas items. The tapes were passed around the inferior vena cava (IVC) and the superior vena cava (SVC). The RHB was established between a 21 F aortic cannula inserted into the main pulmonary artery and two 28 F cannulas inserted into the SVC and IVC. Because of the high PAP, a roller pump was placed between the main pulmonary artery and the SVC and the IVC cannulas to maintain the sufficient blood flow to the pulmonary artery (Fig. 1.). Before cannulation, the cannulas and the circuit were primed with saline to remove any air. Early on RHB, the cardiac output and the mean blood pressure were fluctuating. However, by using low-dose inotrope and crystalloid fluids, this issue was solved, and the mean arterial pressure was maintained at 80 mmHg. The right atrium was opened along the atrioventricular groove; all the vegetation attached to the ICD leads, tricuspid valve, and right atrium wall was removed (Fig. 2.). There was no evidence in favor of valve destruction that needs to be repaired or replaced; therefore, after thorough rinsing, the right atrium was closed. Right heart bypass was discontinued, and de-cannulation was performed as usual. An epicardial temporary pacemaker was implanted for the patient. The ICD generator was removed after closing the chest. The length of operation was 13 minutes. The patient was extubated shortly thereafter and transferred to an intensive care unit (ICU). The post-operative period was totally uneventful, and after two days, the patient was transferred to a surgery ward and discharged home on the fourth post-operative day. The patient was referred to an electrophysiologist for further investigations.

## Discussion

Lead-related infective endocarditis is a rare but serious complication that occurs in 0.38% to 1% of patients^[^1^]^. The mortality rate is rather high and ranges between 11% and 26.9% based on published literature^[^4^]^. Early treatment is mandatory since the patient’s prognosis deteriorates by any delay in the treatment^[^5^]^. Most authors agree that surgical removal is the best option for the treatment of lead-related infective endocarditis, particularly when the vegetation is over 10 mm^[^1,6,7^]^. Studies show that the rate of mortality and recurrence would be lower when the surgery is performed in comparison to medical treatment alone. The percutaneous removal of the vegetation and cardiopulmonary bypass are among the surgical approaches^[^6^]^. The percutaneous removal of the vegetation is limited by the size of the vegetation and can not be performed for vegetation over 23 mm. The open surgical approach assists the surgeon in removing all the thrombi or infectious material thoroughly and in repairing damaged valvular or paravalvular or intracavity structures^[^8^]^. Although cardiopulmonary bypass has been considered a great achievement in medicine, it has its own disadvantages: cold myocardial ischemia, coagulation, activation of platelets and leukocytes, and the inflammatory response, which is the most important complication and occurs as a result of physiologic conversion, cardioplegic arrest, and extracorporeal circulation during cardiopulmonary bypass^[^9,10^]^. The inflammatory response may result in post-operative pulmonary complication including transient hypoxemia or even more severe complication such as acute respiratory distress syndrome (ARDS). Additionally, the ascending aorta manipulation may result in post-operative cerebral atheroembolism and stroke^[^11,12^]^. Based on the literature, another open surgical approach for the treatment of lead-related infective endocarditis in selected patients is RHB, which is used in this article. Cardioplegic arrest is not needed in this technique, and a less post-operative inflammatory response is expected due to the absence of long tubes, an oxygenator, and aortic clamping. Moreover, autologous oxygenation decreases the risk of thromboembolism, maintains coagulation, and minimizes hemolysis^[^13^]^. In that case, the compliations of the cardiopulmonary bypass would be prevented, and at the same time, the patient would benefit from the advantages of the open surgery. In order to find out if the patient is suitable for this approach, a preoperative echocardiography is necessary to study the intracardiac anatomy and evaluate the presence of any connection between the right and left heart including patent foramen ovale (PFO) or septal defect. The presence of PFO anomaly has the risk of air embolism; therefore, it should be detected and addressed even with a very short period of clamping the aorta. Carbon dioxide insufflation is useful to prevent air embolism. Any connection between the right and left heart causes a large amount of blood bypass and fills the surgical field in the right heart. Although a high PAP has been mentioned as a contraindication of right heart bypass, in this article, we solve this problem by using a roller pump. The pump pushes the blood from the SVC and the IVC to the pulmonary artery and helps the blood flow to overcome the high pressure of the pulmonary artery^[^2^]^. Recovery is rapid in this technique, and intensive care unit stay is less than a day. However, this technique is not recommended in hemodynamically unstable patients and long complex operations. A careful attention must be paid to the patient’s oxygen saturation and central venous pressure (CVP), particularly during the off-pump method, as a high CVP could result in neurological damage^[^13^]^.

## Conclusion

Right heart bypass could be a less complicated, safer, and cost-benefit alternative to conventional cardiopulmonary bypass in the case of right-sided heart problems. This technique allows effective decompression of the right heart and adequate oxygenation without post-operative complications, usually seen in routine cardiopulmonary bypass.

## Data Availability

The authors confirm that the data supporting the findings of this study are available within the article.

## Research registration unique identifying number (UIN)

The article is a case report.

## Patient perspective

I am delighted that with the help of the surgeons, I have been able to overcome that hard condition, and now, I am eager to share my experience to help with faster diagnosis and better treatment for similar cases.
